# Modelling collective motion based on the principle of agency: General framework and the case of marching locusts

**DOI:** 10.1371/journal.pone.0212044

**Published:** 2019-02-20

**Authors:** Katja Ried, Thomas Müller, Hans J. Briegel

**Affiliations:** 1 Institut für Theoretische Physik, Universität Innsbruck, Innsbruck, Austria; 2 Department of Philosophy, University of Konstanz, Konstanz, Germany; Texas A&M University System, UNITED STATES

## Abstract

Collective phenomena are studied in a range of contexts—from controlling locust plagues to efficiently evacuating stadiums—but the central question remains: how can a large number of independent individuals form a seemingly perfectly coordinated whole? Previous attempts to answer this question have reduced the individuals to featureless particles, assumed particular interactions between them and studied the resulting collective dynamics. While this approach has provided useful insights, it cannot guarantee that the assumed individual-level behaviour is accurate, and, moreover, does not address its origin—that is, the question of *why* individuals would respond in one way or another. We propose a new approach to studying collective behaviour, based on the concept of *learning agents*: individuals endowed with explicitly modelled sensory capabilities, an internal mechanism for deciding how to respond to the sensory input and rules for modifying these responses based on past experience. This detailed modelling of individuals favours a more natural choice of parameters than in typical swarm models, which minimises the risk of spurious dependences or overfitting. Most notably, learning agents need not be programmed with particular responses, but can instead develop these autonomously, allowing for models with fewer implicit assumptions. We illustrate these points with the example of marching locusts, showing how learning agents can account for the phenomenon of density-dependent alignment. Our results suggest that learning agent-based models are a powerful tool for studying a broader class of problems involving collective behaviour and animal agency in general.

## Introduction

Collective behaviour is a wide-spread phenomenon in biology: fish school to reduce drag, large herbivores gather in herds to avoid predation, bees distribute the task of locating a new nesting site, and even bacteria employ collective decision-making mechanisms in certain circumstances (for an overview, see e.g. [1, 2]). In an effort to understand the dynamics of such swarms, one may ask how the collective effects arise from large numbers of individuals that independently follow simple behavioural rules. The emergence of collective motion is commonly studied using self-propelled particle (SPP) models, wherein individuals are reduced to featureless points that change their motion in response to others’ according to a fixed set of rules [3]. Notably, these rules are simply *assumed* to take a particular form, at most changing (possibly even discontinuously) as a function of some parameter, which may be estimated from experimental data [4–14]. In the present work, we propose a more comprehensive framework for modelling collective motion, based on the notion of *learning agents* (or simply ‘agents’, for short): entities that interact with their environment via *explicitly modelled perceptions and actions*, endowed with an internal mechanism for deciding how to respond, and capable of *adapting* those responses based on an individual history of interactions and feedback [15]. (Note that this adaptation is not merely a matter of being equipped with different behavioural programmes that are triggered depending on certain conditions—instead, learning agents are capable of modifying their responses themselves.) By treating members of the swarm as such agents, one can obtain a more detailed and realistic account of how individuals behave—that is, how they respond to perceptual input and, in particular, to the activity of their neighbours. From there, one can then derive interaction rules, if desired. Most notably, our agents are capable of *learning* how to behave, simply by being exposed to an environment that rewards certain outcomes, rather than requiring their interactions to be postulated *ad hoc*.

In current usage in the literature, the term ‘agent-based model’ refers simply to a model that represents the constituents of a swarm as separate entities [1, 10, 14, 16, 17]—in contrast with continuum models, such as [7, 11, 12], or approaches that coarse-grain the behaviour of distinct individuals to reach (approximately) a continuous limit, as in, e.g., [8, 14, 18, 19]. Here we will refer to such models as merely ‘individual-based’, since we propose a strictly stronger notion of what constitutes an agent. This notion is grounded in philosophical considerations on the nature of agency, but it has also proved to be of great practical value in the context of artificial intelligence and robotics.

An agent-based model in our sense firstly describes how the agents *interact* with their environment. For simplicity, this interaction can be divided into turns, with the agent alternately receiving information from the environment in the form of *percepts* and responding to this by *actions*. The idea of modelling an agent’s perception explicitly proves particularly useful in the study of collective behaviour in animals, since it allows one to explore which stimuli individuals must be able to perceive in order to account for the observed behaviour.

The second part of an agent-based model describes the agents’ internal processes, by which they deliberate on the received percepts and choose actions in response. (Note that we do not aim to detail the biological functioning of sensory organs or neural processes in any particular species, which is an intriguing research topic in its own right, but instead focus on the processing of information on a more abstract level.) These processes are modelled in the simplest possible terms, both to reflect the simplicity of the abstract information-processing capabilities that many real life-forms use to respond to environmental stimuli, and to allow for an easier interpretation of the results obtained via the model. However, it is essential that the agents’ internal processes be sufficiently complex to support *memory*, in the sense of some record of the agent’s past interactions with its environment. This enables the agent to gradually develop its own, individual behavioural tendencies—in short, to learn—which is what sets it apart from mere pre-programmed automata. The learning process is guided by *rewards*, which, at an abstract level, are generally cues provided by the environment to indicate that some desirable condition (such as completing a maze) has been met. Upon receiving a reward, the agent updates its deliberation mechanism, reinforcing those responses that led to this outcome. The concrete framework of agency that we use, termed Projective Simulation (PS) [15], is introduced in more detail in the section ‘Learning-agent-based models’.

We illustrate the principles of learning-agent-based modelling with the example of locusts: short-horned grasshoppers which form swarms, capable of consuming hundreds of tonnes of food per day and threatening the livelihood of entire regions. Due to this considerable ecological and socioeconomical impact, the collective motion of locusts has been studied extensively [20]. One of the most notable features of the motion of marching locust nymphs is density-dependent alignment (see e.g. [17, 21]): at low densities, the motion is completely disordered, whereas at higher densities, individual movement is strongly aligned in a single direction, which remains constant for stretches of time. This directed movement is punctuated by sudden changes in the group’s direction, which occur less frequently as density increases. In the following, we will consider this phenomenon as a case study in modelling.

Density-dependent alignment has the advantage that it can be observed in a greatly simplified laboratory setting: locusts are confined in a narrow circular arena, which constrains them to move (approximately) in one dimension. (A formal description of the scenario faced by the locusts in given in the section ‘The environment model’.) This considerably simplifies the mathematical description of the phenomenon: in one dimension, the swarm’s alignment can be summarised by a single parameter *z* ∈ [−1, +1], given by the group average of the direction of motion. Plotting the time-dependence *z*(*t*) reveals a striking visual signature of the different regimes of collective motion, as one can see, e.g., in the experimental data reported in [21], and also in the results of our own simulations, in section ‘Results’.

In the present work, we show how an agent-based model of marching locusts can account for this effect, as well as other experimentally observed features of locusts’ collective motion. Moreover, we will show how learning agents can develop on their own a pattern of individual-level behaviour that can explain their collective dynamics.

## Learning-agent-based models

We begin by introducing the conceptual framework of learning agents, followed by a formal, mathematical formulation. We then illustrate the use of agent-based models with a task environment that models the dynamics of locusts swarms.

### Defining agency

Given the central role that learning agents play in our model, we start with some fundamental considerations of what sets such agents apart and how they can be modelled formally. The first essential element of an agent-based model is a description of how the agent interacts with its environment. On the one hand, the agent obtains information about the environment in the form of a *percept*, which we denote *s*. In response to a percept, the agent chooses an *action* *a*, which in turn changes the state of the environment. One turn of the interaction therefore consists of perception, deliberation, action, and in general a reward, as described below.

The second part of an agent-based model describes the agent’s internal processes, which enable it both to deliberate on the received percept, choosing an action, and to eventually update the mechanism of deliberation. Research in both neuroscience and artificial intelligence has generated a variety of proposals for how these internal processes could work, some of which can account for remarkable feats of learning and intelligent behaviour. However, the more sophisticated of these models generally require relatively powerful universal computers to run, and therefore cannot be considered accurate models of how biological entities—whose neural architecture is, at the most fundamental level, relatively simple—can exhibit such behaviours. We favour instead a simpler model of the agent’s internal processes, one that does not presume general-purpose computational capabilities, but which is instead supported by the natural dynamics of the physical system that embodies these processes. In the case of locusts in particular, this restriction to relatively basic internal processes seems realistic. Moreover, even if the actual agent is, arguably, a more complex organism, simpler models quite generally yield more easily interpretable results.

A particularly important feature of the agent’s internal processes is its capacity to retain a memory of past interactions with the environment, since without this, there could be no learning. In order for us to assess whether an agent possesses this type of memory and learning ability, it helps if the agent has a clear goal to achieve. For example, one can easily conclude that an animal is learning if it modifies its behaviour in such a way as to navigate a maze—and ultimately reach a reward—more quickly. In the case of artificial agents, the reward is reduced to an abstract variable, but it nevertheless plays an essential role in the learning process: upon receiving a reward, the agent updates its deliberation mechanism, reinforcing those responses that eventually lead to a reward. Over time, these changes improve the agent’s chances of earning rewards, and thus the agent learns.

### A formal model of learning agents

A concrete proposal for an agent of this type was introduced by Briegel and De las Cuevas in [15]. (Specifically, reference [15] presents a broad proposal for a single agent, introducing several abstract learning tasks as well as features that enable the agent to handle them, such as reflection and association, whereas the present work proposes the use of interacting groups of simpler agents of this type for modelling collective behaviour.) The mathematical and conceptual framework developed therein is termed Projective Simulation (PS), after the defining feature of the agents: they can effectively project themselves into conceivable futures and simulate likely consequences of their actions before realising them. The model has been used, for example, in advanced robotics in the problem of complex skill learning [22]. While such high-level capabilities are not necessary for the present work, a simpler variant of the PS agent does promise to be an interesting model for individuals that can perceive their neighbours, respond to their presence according to certain—adaptable—rules and ultimately exhibit collective dynamics.

**Fig 1:**
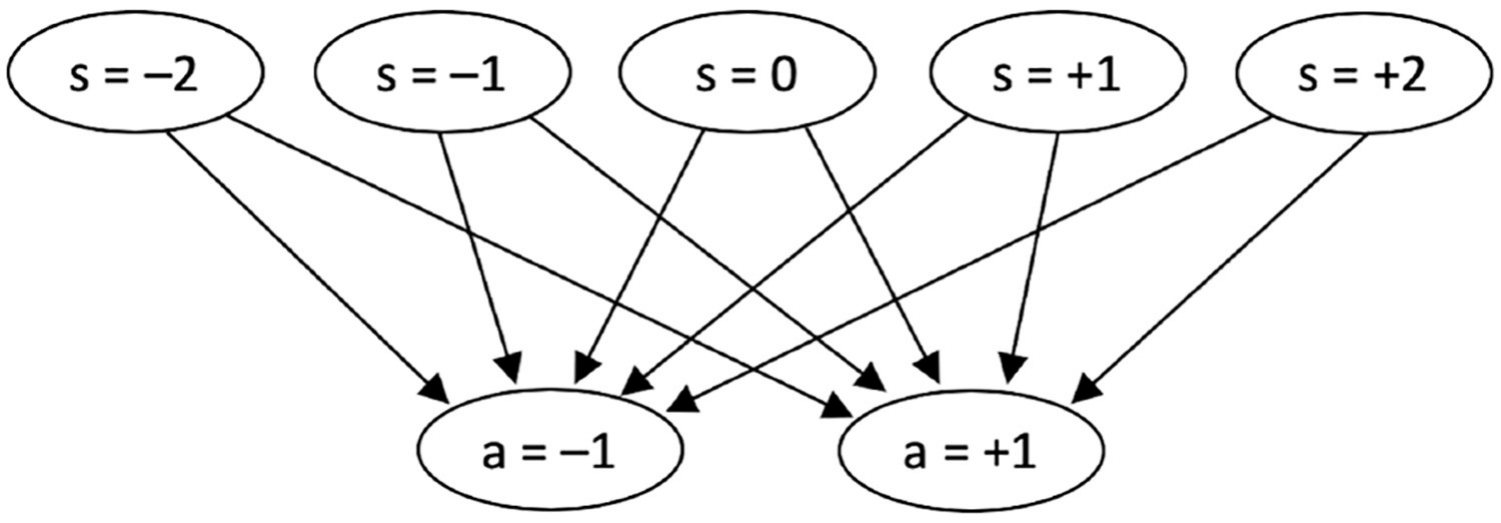
Simple, two-layer ECM (episodic and compositional memory) network for navigating the one-dimensional environment of our locust simulation. The first layer of nodes represent percepts, which encode the net relative flow of nearby conspecifics *s* ∈ {−2, −1, 0, +1, +2}, whereas the second layer represent actions, of turning (*a* = −1) or continuing in the same direction (*a* = +1).

The interaction of a PS agent with its environment follows the scheme introduced above. Its memory structure, which supports the internal deliberation process, is based on snippets of previous experiences and actions, termed *clips*. Transitions between clips are interpreted as the agent recalling or simulating sequences of events that have been rewarded, for instance “percept: predator” → “action: flight”. Formally, one can represent the clips as vertices and the possible transitions as edges of a directed graph, which is termed the *clip network* or episodic and compositional memory (ECM, see [15]). Simple agents possess only two types of clips, representing remembered percepts and actions, which are arranged in a two-layer network, as shown in Fig 1. In general, more sophisticated agents may also possess intermediate clips that represent neither pure percepts nor actions, but this added complexity is not required for the present task.

The deliberation process of a PS agent is realised as a random walk over the clip network: that is, the sequence of internal states that the agent passes through, going from the initial percept to the ultimate action, is represented as a sequence of clips being activated, with the activation jumping from one clip to the next along the (directed) edges of the network. The probabilities of jumping from one clip to the next are governed by the *hopping matrix h*, which stores information about past interactions in the form of time-dependent *h-values* $h_{ij}^{\left(t\right)}\geq 1$ associated with each edge *i* → *j*. In the standard PS framework, the probability of the excitation hopping from a clip *i* to a clip *j* is determined from the *h*-values by simple normalisation,

$$P^{\left(t\right)}(j|i)=\frac{h_{ij}^{\left(t\right)}}{\sum_{k}h_{ik}^{\left(t\right)}}.$$ (1)

Once an action clip is reached, the walk terminates and the agent realises the corresponding action. If a reward is received, in the simplest case, its numerical value *R* is added to the entries in the *h*-matrix that correspond to the transitions used in the random walk, thereby making these transitions more likely in the future. (The value *R* = 0 therefore corresponds to no reward being received).

More generally, an agent may need to adapt to a variety of learning scenarios that present different challenges, such as reward schemes that change over time, or delayed rewards. By refining the above rules for deliberation and updating, it is possible to endow the agent with the ability to handle several more general classes of problems without actually tailoring it to particular tasks, as described in [15, 23]. One refinement that is relevant for the present work addresses the possibility of an environment that changes with time. In such an environment, agents should not commit deterministically to any one action, since it may cease to be rewarded at some point, but they should in fact have the ability to reduce the associated probability. This can be achieved by damping the *h*-matrix by a factor of 1 − *γ* at each time-step, in a process termed forgetting: if *γ* = 0, the agent does not forget at all; if *γ* = 1, it forgets immediately and is therefore incapable of learning over time. (Note that, while *h* is updated during the learning process, so-called meta-parameters like *γ* are fixed properties of the agent).

Combining the mechanisms of reinforcement and forgetting, one can write a single update rule, with two cases depending on whether the transition was used in the deliberation process or not: the *h*-values after the *t*-th interaction are given by

$$h_{ij}^{\left(t+1\right)}-1=(1-\gamma )(h_{ij}^{\left(t\right)}-1)+\{\begin{array}{cc} R^{\left(t\right)} & \text{if}\;\text{used},\\ 0 & \text{if}\;\text{unused,} \end{array}$$ (2)

where *R*^(*t*)^ denotes the reward received at turn *t*. (One may note that damping drives the *h*-values towards unity, rather than zero. This prevents pathological behaviour when normalising as per Eq (1) and ensures sufficient flexibility in scenarios with changing tasks, as detailed in [15]).

As far as its learning dynamics is concerned, projective simulation belongs to the class of reinforcement learning models [23]. Indeed, one could also use standard reinforcement learning algorithms, such as Q-learning or SARSA [24], to study the learning process that allows us to derive how individuals behave and interact. One of the benefits of using the PS model is that it offers an explicit and straightforward account of the agent’s deliberation dynamics.

### The environment model

As described in the Introduction, the world in which the agents move is taken to be one-dimensional and circular, with the ends identified so that the agents can cross in both directions (periodic boundary conditions). For the purposes of the simulation, the world is divided into *W* discrete spatial blocks. The agents move at a fixed speed of one block per time-step and can pass through each other (that is, we do not restrict the number of agents occupying the same position). A second fundamental parameter in our model is the sensory range *r*, which gives the distance up to which a given agent can perceive others. Together with the size *W* of the world, this defines the number *B* = *W*/2*r* of non-overlapping ‘neighbourhoods’ or ‘bins’, which turns out to be a relevant parameter for the collective dynamics. Finally, we let *N* denote the number of agents in this world. An overview over the relevant parameters is given in Table 1.

**Table 1:** Overview of parameters describing the environment and the agents, along with numerical values used in simulations.

Environment parameters	
*W* = 80	world size (in unit steps)
*r* = 5	sensory range (in unit steps)
*B* ≡ *W*/2*r*	number of non-overlapping neighbourhoods
*N* ∈ {10, 40, 70}	population size
Agent parameters	
*d* ∈ {1, 5, 30}	decisiveness (in Eq (3), for non-learning agents)
*γ* ∈ {0, 0.002}	forgetting (only for learning agents)

In our model, the percept *s* available to an agent is the net flow of individuals within range *r* relative to the agent, that is, the number of neighbours going in the same direction as the agent minus those going in the opposite direction,distinguishing only absolute values up to two: *s* ∈ {−2, −1, 0, +1, +2}. (Although we give a formal, mathematical definition of the percept, it is not plausible that the locust explicitly counts neighbours and subtracts their numbers to obtain this information. A more realistic interpretation is that, for example, the visual flow provides information about *s*.) In response to this percept, an agent can choose to either turn around (*a* = −1) or continue in the same direction (*a* = +1). Notice that the agents take their actions one after another, a fixed sequence, so that, while one individual is deliberating, none of its neighbours will change direction. The reward scheme, for this first exploration of density-dependent alignment, is deliberately simplified: an agent is given a reward *R* = 1 if, at the end of its turn (i.e., after taking its action), it is moving in the same direction as the majority of its neighbours.

It is worthwhile to highlight two subtle differences between this model and the treatments commonly seen in the literature, such as [1, 4–8, 10–12, 14, 17–19]. For one, existing models almost universally prescribe synchronous updating, meaning that all individuals in the group apply their update rule at the same time. This can simplify the mathematical treatment (for example when taking the continuum limit [7, 11, 12]), but it has been pointed out that the implicit assumption of synchronous updating may not be the most realistic in many scenarios, and that it may inadvertently distort or constrain the types of collective dynamics that arise from the model [25, 26]. Indeed, contemplating a group of locusts, it seems extremely unlikely that all individuals would update their headings simultaneously, and much more realistic that changes would be made by single individuals at a time. In terms of modelling, reinforcement learning (as described here) is more naturally suited to such asynchronously updating environments, since simultaneous updates due to the actions of several individuals make it more difficult for each agent to assess the consequences of its own choices. However, it is possible to modify the present model to include synchronous updates, should this be deemed more appropriate for a given scenario.

A second difference of the present environment model is the role of the sensory range *r*. While many SPP models define one or more zones that deterministically imply particular interactions (such as attraction, alignment or avoidance, see e.g. [4, 6, 12]), the parameter *r* in our model merely constrains what data are *available* to the agent, but without determining a priori that conspecifics within range *r* will have any particular effect on the focal individual. Instead, the appropriate responses to perceiving conspecifics at various distances (or bearings, for agents with more fine-grained perception) are determined during the learning process.

### Agents with fixed behavioural dispositions

Fig 1 depicts the ECM used by agents in our simulation of locusts. Since all edges in the network connect percepts directly to actions, the *h*-values can be indexed as $h_{s,a}^{\left(t\right)}$, forming a 5 × 2 matrix. For the purpose of studying the SPP limit of our model, agents are pre-programmed with a set of fixed *h*-values that lead to successful behaviour. For the alignment task described above, a suitable choice is

$$\begin{array}{cc} & h_{+2,+1}^{\left(t\right)}=h_{-2,-1}^{\left(t\right)}=1+d\\ & h_{+1,+1}^{\left(t\right)}=h_{-1,-1}^{\left(t\right)}=1+\frac{1}{2}d\\ & h_{s,a}^{\left(t\right)}=1\;\;\text{otherwise}, \end{array}$$ (3)

with a single parameter, *d*, controlling how decisively the agent turns around when facing opposition (*s* < 0) or keeps its direction when it is aligned with its neighbours (*s* > 0).

We stress that the auxiliary parameter *d* is added only to emulate a conventional SPP-type model; it embodies an *assumption* about how the individuals interact. In the case of group alignment in one dimension, it is relatively easy to guess a set of response probabilities that can account for the observed collective dynamics, but as one proceeds to modelling more sophisticated systems, this becomes increasingly difficult. In those cases, learning must be used, meaning that agents are initially endowed only with a ‘neutral disposition’ (all $h_{s,a}^{\left(0\right)}=1$) and then left to discover the appropriate response probabilities themselves.

Note that an agent whose memory is described by the *h*-matrix in Eq (3), when presented with the percept *s* = 0 (zero net flow of neighbours), may turn or continue with equal probabilities. This behaviour also emerges in a PS agent whose *h*-matrix is not fixed (see section ‘Learning individual-level behaviour’): unless some preference is acquired based on asymmetric rewards, all actions are taken with equal probability. In biological entities, on the other hand, not turning can be considered a default action, being energetically more favourable, and one would expect individuals to turn with probability less than $\frac{1}{2}$. This feature can be included in our model by introducing an ‘inertia’ parameter, which makes $h_{0,+1}^{\left(t\right)}>h_{+2,-1}^{\left(t\right)}$ and consequently $P\left(a=+1|s=0\right)>\frac{1}{2}$. Our simulations indicate that this modification has no discernible effect on the phenomena described in the present work.

## Results

### Density-dependent alignment of agents with fixed response probabilities

The first test of our agent-based model is whether, in the limit of a single, fixed rule for individual-level responses, it can produce different regimes of collective alignment as a function of swarm density. By disabling the learning subroutine in PS agents and instead proposing a fixed form for the *h*-matrix (as described in the preceding section), the agents are effectively reduced to self-propelled particles and we recover an individual-based model. We wish to verify whether, in this limit, our model can account for density-dependent alignment equally well as existing individual-based models.

**Fig 2:**
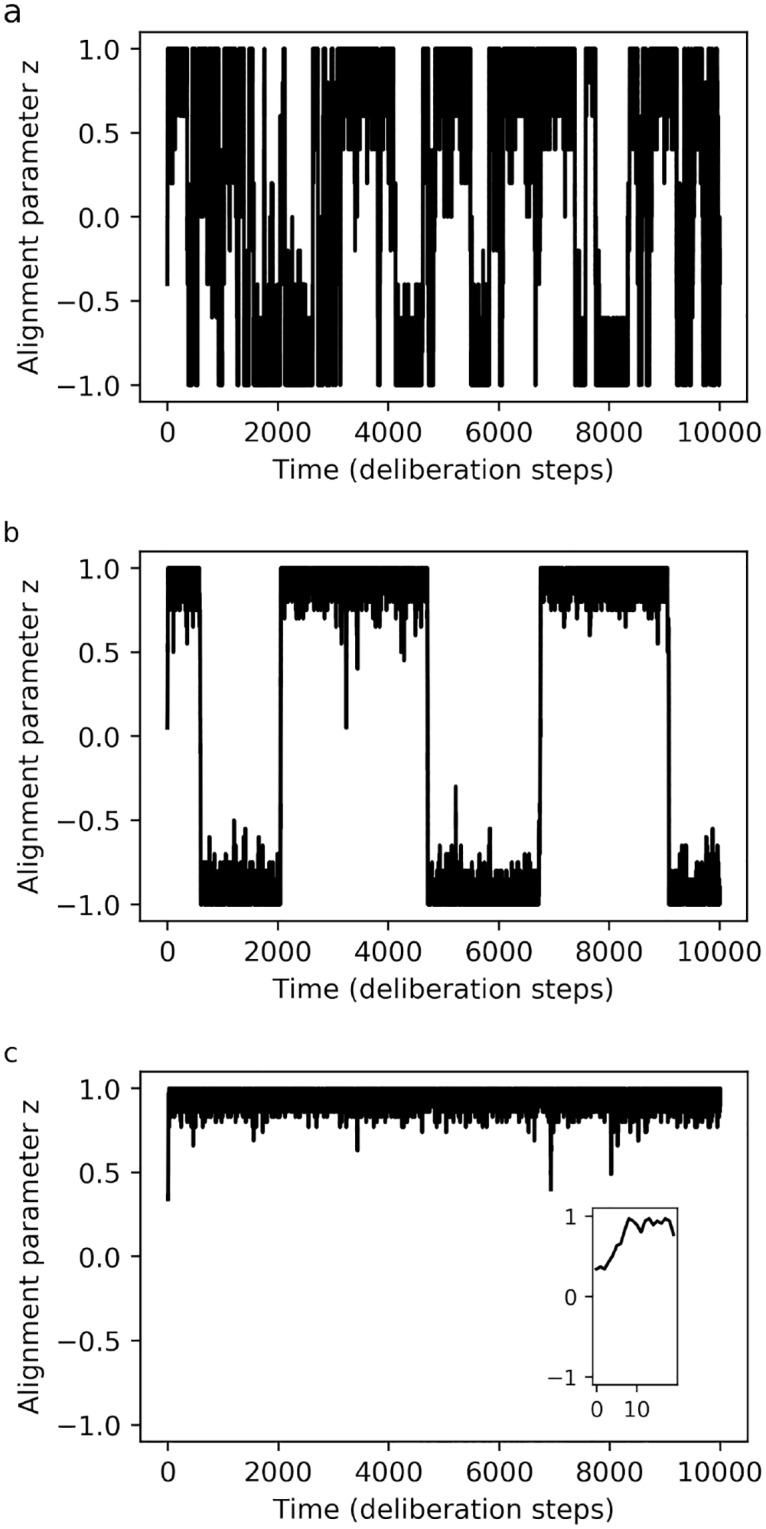
**Different regimes of collective motion for agents with fixed decisiveness, as a function of population size *N* in a world of fixed size *W* = 80**: (a) *N* = 10 (low density): Disordered motion, (b) *N* = 40 (intermediate density): Global alignment with stochastic changes in direction, (c) *N* = 70 (high density): Global alignment, direction is stable over long periods. Plots show the alignment parameter *z* (group average of the direction of motion) as a function of time, with one time-step corresponding to one cycle of interaction and deliberation by the PS agent. The inset in panel (c) shows that, on much shorter time-scales, the group transitions from the disordered (randomised) initial state to the ordered long-term state. Parameters: decisiveness *d* = 30, sensory range *r* = 5 (number of bins *B* = 8).

Fig 2 shows the results of simulations wherein varying numbers of individuals with fixed response probabilities (fixed *d*) are placed in a world of fixed size, with initial positions and orientations distributed uniformly at random. One can see that our model can account for the different regimes of collective dynamics as a function of density that are observed in experimental data (see e.g. [21]). Reproducing this phenomenon is one of the most basic benchmarks for a model of locust motion, and achieving this puts our agent-based model on a par with previous models, both individual-based and continuous. However, we stress that our model does not require any fine-tuning of the individual-level behaviour in order to account for the different regimes of motion, as some individual-based models do.

### Comparison of predictions in terms of the Fokker-Planck equation

While plots of the alignment parameter *z*(*t*) (the group average of the direction of motion) provide an intuitive visual representation of the collective motion, each such plot only depicts a single possible evolution and therefore fails to address important questions about the general features of the dynamics, for instance how strongly aligned the metastable states are and how quickly the system transitions between them. In order to study these statistical properties, we consider the probability distribution *P*(*z*, *t*), whose evolution is described by a Fokker-Planck equation (FPE)

$$\frac{\partial }{\partial t}P(z,t)=-\frac{\partial }{\partial z}[F(z)P(z,t)]+\frac{\partial^{2}}{\partial z^{2}}[D(z)P(z,t)].$$ (4)

The functional form of the two coefficient functions encodes essential features of the group-level dynamics: *F*(*z*) gives the drift velocity (that is, how quickly an element of probability *dP* moves along *z*), governing systematic changes in *z*, while *D*(*z*) governs the diffusion of the probability density, representing noise. This representation of the dynamics has proven widely useful in the study of collective motion, for example for comparing the predictions of various models and analysing the existence and stability of ordered states [14, 17, 19]. In the case of our agent-based model, these functions can be calculated analytically from the individual-level interaction rules, as detailed in S1 Appendix.

**Fig 3:**
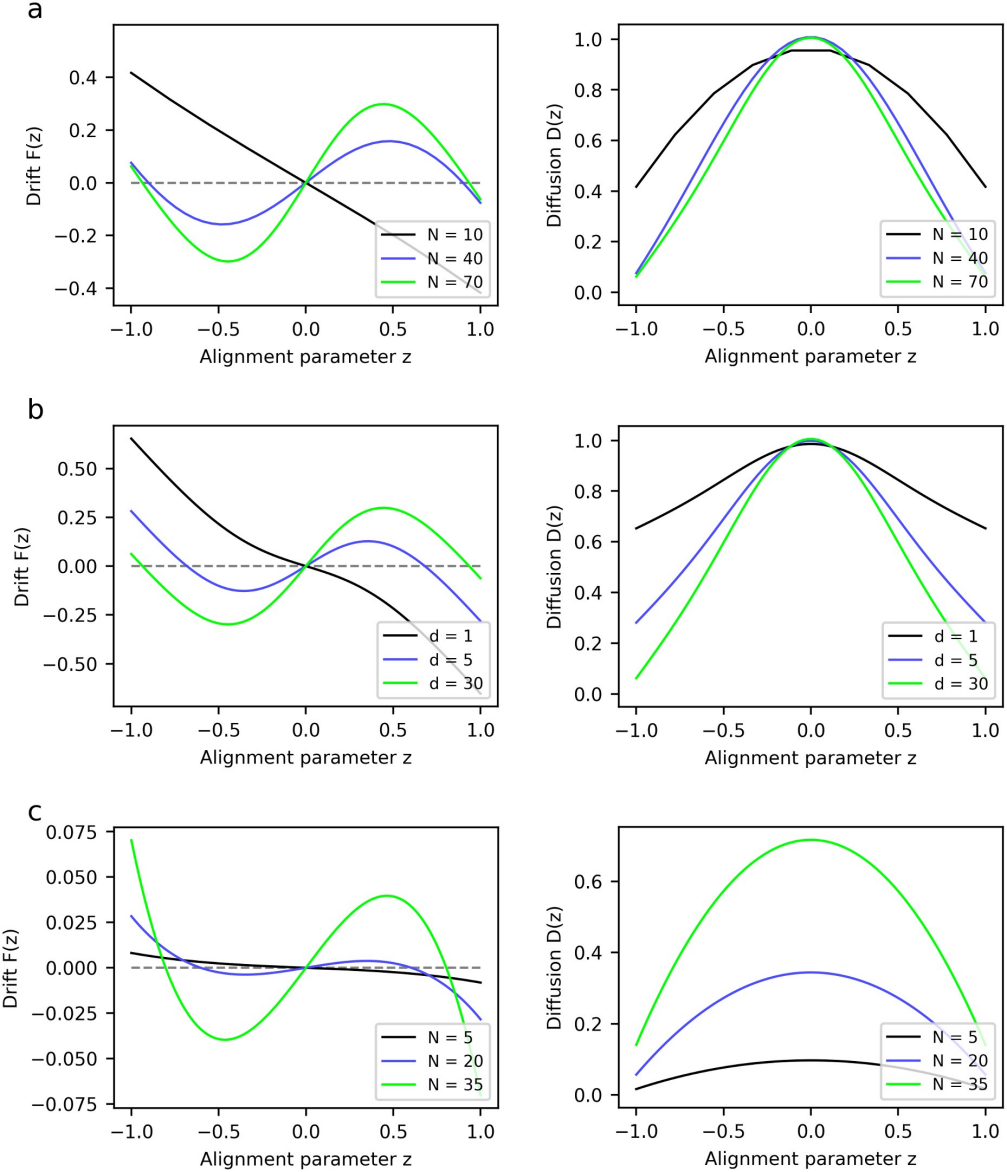
Group-level dynamics, described by the two coefficient functions in the FPE; drift *F*(*z*) (left) and diffusion *D*(*z*) (right). Zeros of the drift function are fixed points, with $\frac{dF}{dz}(z_{0})<0$ indicating attractors, that is, stable equilibria, while $\frac{dF}{dz}(z_{0})>0$ implies unstable fixed points. Outside of fixed points, large-magnitude drift entails quicker transitions between states, while strong diffusion makes the system more likely to leave a given state. We show (a) predictions of an agent-based model with fixed decisiveness *d* = 30 and variable effective density *N*/*B* (number of individuals per neighbourhood), fixing *B* = 8, (b) predictions with fixed effective density (*N* = 70, *B* = 8) and variable decisiveness. Panel (c) reproduces the coefficient functions for drift *F*(*z*) (left) and diffusion *D*(*z*) (right) of Dyson *et al*. [19], which were obtained by fitting experimental data to a model based on transition rates, for different numbers of individuals *N*.

Fig 3a and 3b shows the predictions of our agent-based model for the drift and diffusion coefficient functions of the FPE. We explore the effects of varying two parameters: the effective density *N*/*B* and the decisiveness *d*. For comparison, Fig 3c reproduces the corresponding curves generated by a model fitted to experimental data by Dyson *et al*. [19], for different numbers of individuals *N* confined to an arena of fixed size.

Comparing the essential, qualitative features of the drift and diffusion functions, one can see that the predictions of our model are in line with experimental data. At low densities, there exists a single stable state (with zero drift and relatively high diffusion): this is the completely disordered state, with the alignment parameter *z* = 0. At higher densities, two additional stable fixed points arise at *z* ≈ ±1, which correspond to ordered collective motion in one direction or the other. At the same time, the disordered state becomes unstable, since drift tends to move the system away from *z* = 0. One can see that, as was the case for the previous analysis in terms of the dynamics *z*(*t*), if one takes the limit of fixed individual-level responses, then the agent-based model reproduces the predictions of existing SPP models. (A comparison of several theoretical models of marching locusts in terms of the FPE coefficient functions can be found in [17]).

However, our model also allows us to go beyond the effects of density alone and explore the influence of a second degree of freedom, namely the decisiveness governing responses at an individual level. Quite generally, if one wishes to provide an account of collective dynamics as arising from individual behaviour, then it is desirable that some parameter(s) governing that behaviour feature prominently in the model. The effects of the decisiveness parameter *d* in our model are illustrated in Fig 3b, and one can see that they are are qualitatively very similar to the effects of the density parameter discussed above. This implies that, while the different regimes of collective motion can be triggered by changes in density, they could equally well be due to changes in the parameters governing behaviour at an individual level. To our knowledge, there have been no experiments to date that explore this possibility.

When comparing plots of *F*(*z*) and *D*(*z*) from different sources, one should note that the scaling of the ordinate axes reflects the scaling of the time variable used in the FPE. In the experiments discussed and analysed in Fig 3c, time is measured in steps of 0.8*s* and 0.2*s*, respectively, for drift and diffusion. In the agent-based model, on the other hand, the natural unit is the deliberation time-step, that is, the time-scale on which individuals interact. If the relation between these two time-scales is unknown, then the predictions of the two models can only be meaningfully compared up to a scale factor. However, it is in principle possible to determine this scale factor by comparing the drift and diffusion rates predicted for equivalent scenarios in the two models. This will provide a testable prediction of the time-scales on which locusts respond to the movement of surrounding conspecifics, which should prove a useful parameter to estimate. This is one possible direction for future work.

The most striking difference between the experimental data reproduced in Fig 3c and the predictions of our model concerns the scaling of the diffusion function with the number of individuals, which can be seen most easily at *z* = 0: in the results of Dyson *et al*., *D*(*z* = 0) grows approximately linearly with *N*, whereas in our model, it is approximately independent of *N*. (A similar scaling with *N* can be observed in the drift function *F*(*z*), although assessing the scaling of a third-order polynomial is less straightforward.) However, it is unclear whether this difference in scaling factors is a symptom of an actual discrepancy or merely an artefact: as we pointed out above, the choice of time units directly affects the scaling of the drift and diffusion functions, and Dyson *et al*. do rescale their time variable by a factor of *N* at one point, which could account for the differences observed here. We were unable to locate any other sources that present experimental results in a form that could be translated to FPE coefficients in order to clarify this point. However, the predictions of our model are in line with those of other theoretical models, specifically those of Czirók and Buhl [17], who find *D*(*z* = 0) to be largely independent of the density, just as it is in our model.

### Learning individual-level behaviour

In the previous two subsections, we have argued that agent-based models offer a more detailed and potentially more realistic account of the perceptions, responses and internal processes of individuals in a swarm than traditional individual-based models. We now turn to the second major advantage of agent-based models: the fact that there is no need to postulate how agents interact, since they can learn the required behaviour by themselves. In our example of locusts, we can now drop the assumption of a fixed *h*-matrix (parametrised by the decisiveness *d*, as shown in Eq (3)). Instead, learning agents are initially given only ‘neutral’ dispositions, along with rules that allow them to learn by themselves.

**Fig 4:**
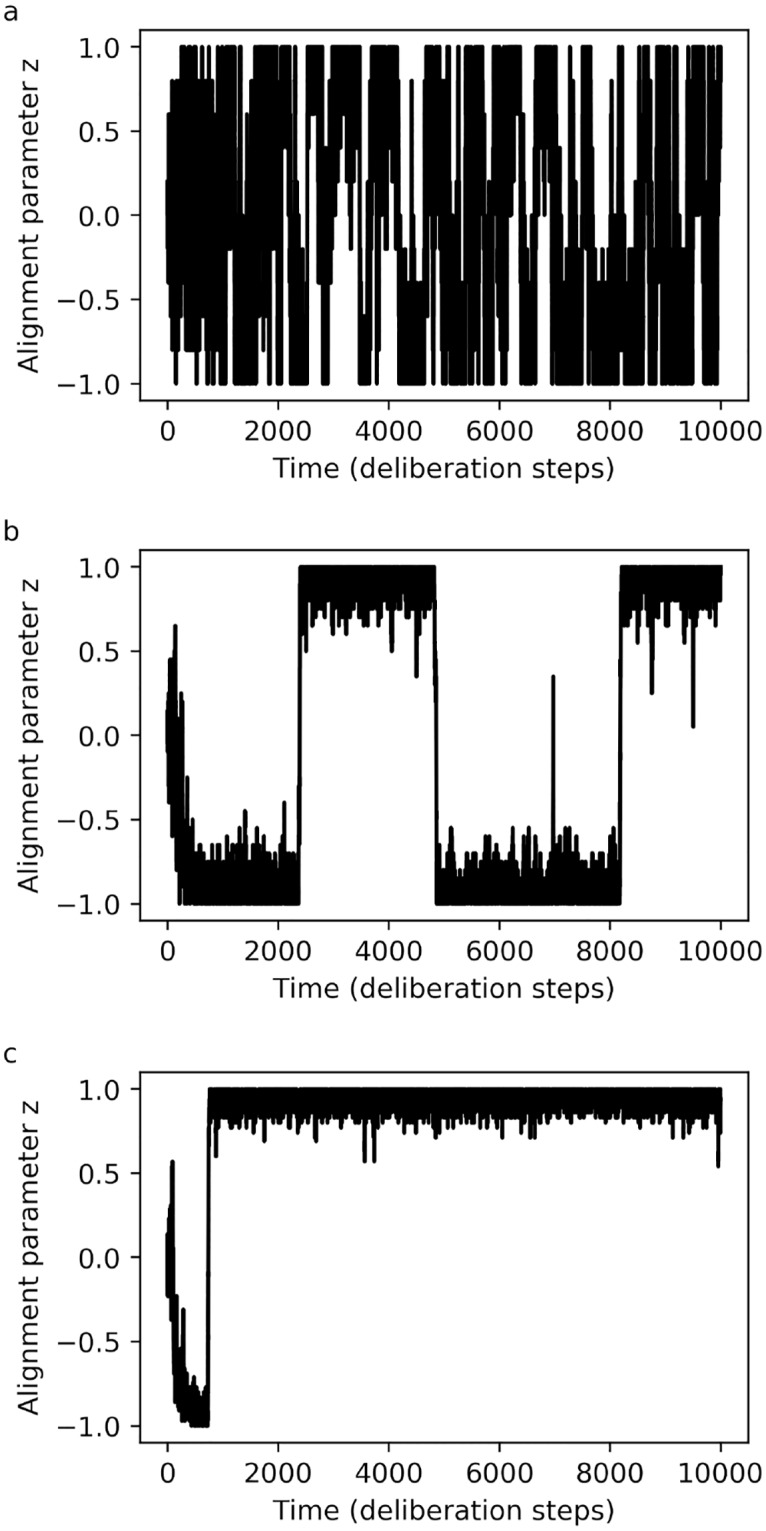
**Global alignment *z*(*t*) for learning agents**: (a) low density (*N* = 10) leads to disordered motion, (b) intermediate density (*N* = 40) leads to alignment with periodic switching, and (c) high density (*N* = 70) leads to long-term alignment. The appearance of well-defined regimes of collective dynamics is preceded by a transient learning period, during which agents’ individual behaviour is still changing. Parameters: world size *W* = 80, sensory range *r* = 5 (number of bins *B* = 8) and forgetting parameter *γ* = 0.002.

**Fig 5:**
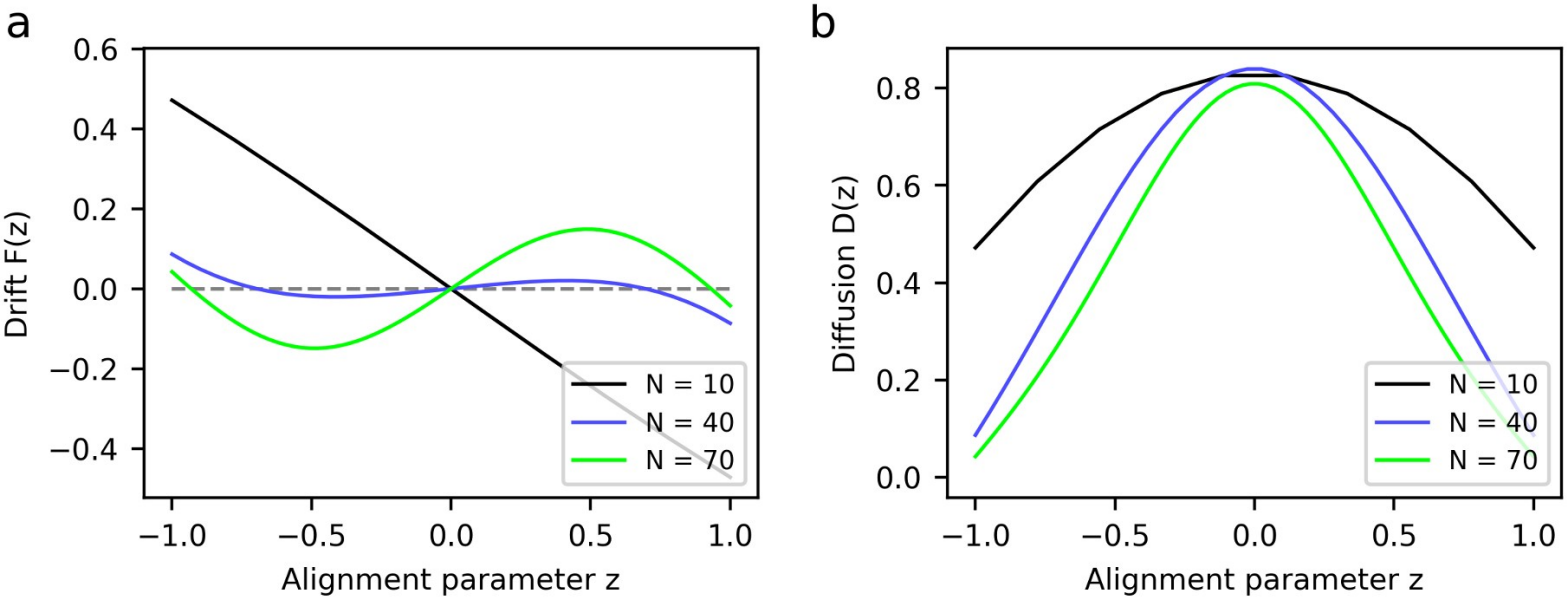
**Group-level dynamics of learning agents, described by the two coefficient functions in the FPE**: (a) drift *F*(*z*) and (b) diffusion *D*(*z*). Note how the population size *N* results in qualitatively differences in the forms of the drift function, which in turn implies different regimes of collective motion. The group dynamics are based on the behaviour developed by learning agents after 10^4^ interaction steps, averaged over the ensemble of agents. Parameters: world size *W* = 80, sensory range *r* = 5 (number of bins *B* = 8) and forgetting parameter *γ* = 0.002.

We find that, at the level of collective motion, our learning agents successfully reproduce the phenomenon of density-dependent alignment, as can be seen in Fig 4 and by the drift and diffusion functions in Fig 5. This shows, for one, that it is possible to learn a set of individual-level behavioural rules that can account for the dynamics of marching locusts.

A closer analysis of Fig 4 reveals that the learning-agent model also captures an additional feature: a transient regime, tracking changes in the collective dynamics that arise while the individual-level behavioural dispositions (technically, the response probabilities) are still being modified noticeably. Note that this is different from the transients normally found in SPP models—and also in the fixed-disposition version of our own model, in Fig 2 –, which are due simply to the random initial positions and orientations of the particles. By contrast, the transient in Fig 4 is due to the fact that the agent’s *response probabilities* take some time to equilibrate. While this is suggestive of the solitarious-gregarious phase transition observed in locusts, one may note that the transition seen in our simulations begins with agents that respond at random, and not as solitarious locusts would. One could change this by designing a second reward scheme that favours the behaviour typically shown by solitarious locusts, training agents initially with that scheme and later switching to the one we use here. More fundamentally, when real locusts undergo a phase transition, they are not learning a new set of behavioural rules, but rather switching to a different set of rules that had already been acquired over the course of evolutionary adaptation, using suitable stimuli as cues to trigger the switch. In principle, learning agents can also develop this more complex behaviour, learning different sets of responses that are applied depending on additional cues. In general, considering the central role that phase transitions play in the formation of swarms and plagues, having a theoretical model for predicting collective dynamics which naturally accommodates changing individual behaviour could be extremely useful.

**Fig 6:**
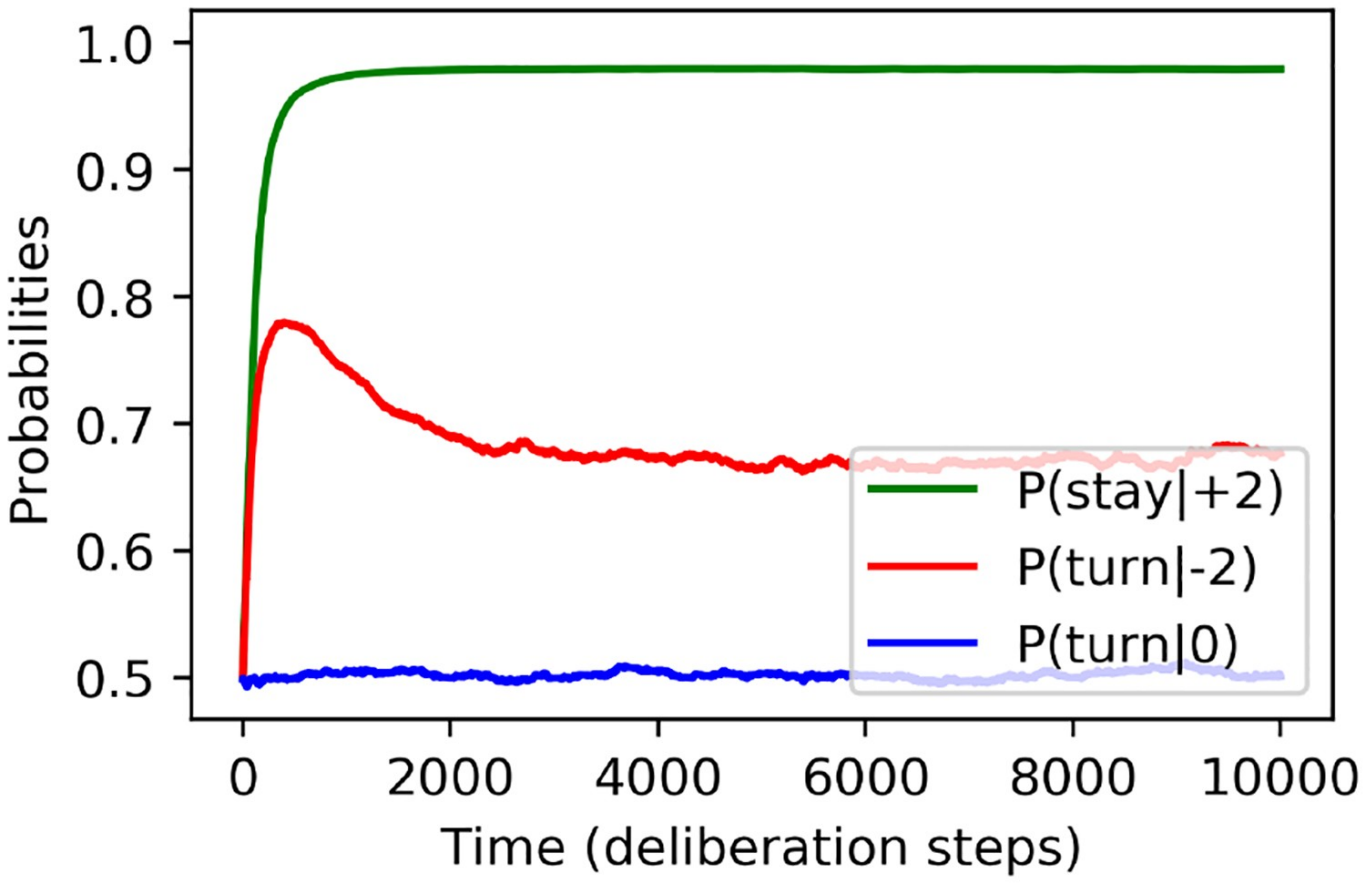
Learning individual responses: Probabilities of various responses to percepts as a function of time (measured in discrete interaction steps), averaged over a group of *N* = 40 agents. Curves depict the probabilities of individuals (green) maintaining their direction when perceiving a net flow in the same direction they are moving, (red) turning around when facing a net flow in the opposite direction, and (blue) turning around when perceiving zero net flow. A rational agent should develop high probabilities for the first two, while the last probability is expected to remain at $\frac{1}{2}$. Parameters: world size *W* = 80, sensory range *r* = 5 (number of bins *B* = 8) and forgetting parameter *γ* = 0.002.

Having verified the predictions at the collective level, we now focus on the key feature of learning-agent-based models, namely the possibility of deriving how *individuals* respond to various stimuli. In order to track this learning process, we plot how agents respond—probabilistically—to different perceptions, as a function of time. Fig 6 shows that our learning agents develop the expected responses to different percepts given feedback from the environment. In fact, the learning process proceeds quite quickly, over time-scales of the order of 10^2^ time-steps, which is not unexpected given the small spaces of percepts and actions in our model.

One may note in Fig 6 that learning agents are more likely to maintain their direction if they are already aligned with the majority of their neighbours (percept *s* = +2) than they are to turn around when facing opposition (*s* = −2). This is due to a simple statistical effect: as the members of the group become more likely to align themselves with the majority of their neighbours, percepts indicating a net flow in the opposite direction are encountered less frequently. Consequently, the decisiveness with which they respond, which is determined by the balance of reinforcement and forgetting, is smaller than for more frequently encountered percepts. (It was established in [15] that stronger forgetting, which is essential for adaptation to changing environments, simply lowers the asymptotic probability of taking the rewarded action while leaving the initial learning curve largely unchanged, for a wide range of values *γ*. At the same time, stronger forgetting leads to larger statistical fluctuations in that probability, as one can also see in Fig 6.) Meanwhile, the percepts representing a net flow in the *same* direction as the agent are received increasingly frequently, so that the correct response (namely to keep going in the same direction) is reinforced more often. It follows that the decisiveness with which the agent responds to these two types of percepts becomes noticeably different with time. In short, the different asymptotic response probabilities reflect the fact that, in the scenarios agents encounter in our simulations, how they respond to *s* = +2 is more relevant for their overall success. (One can modify the task environment to confront agents with both *s* = +2 and *s* = −2 in a more balanced manner, for instance by periodically randomising the positions and orientations of all agents. This does reduce the difference between the asymptotic response probabilities for the two percepts).

It is interesting to note that, even though the learned behaviour is different from the fixed rules assumed in the section ‘Density-dependent alignment of agents with fixed response probabilities’ (formalised by Eq (3)), the collective dynamics are the same. This shows that the collective dynamics (at least those features which we have analysed) are robust under certain *changes* to the individual-level behaviour, such as different values of the decisiveness with which agents respond to two types of percepts.

**Fig 7:**
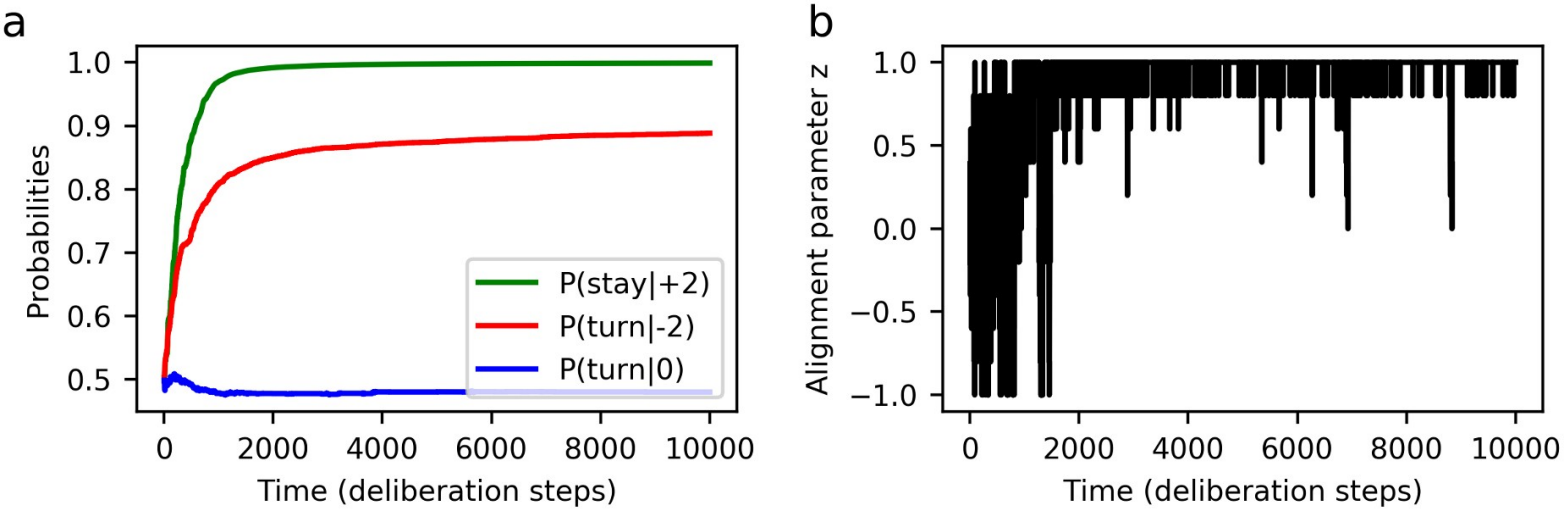
**Agents that learn without forgetting (*γ* = 0)**: (a) Response probabilities grow towards unity. (b) The collective motion tends to be strongly aligned, with *z*(*t*) fixed at ±1 for long times. This is noteworthy since the scenario presented here has relatively low density, with the same parameter values for *N* and *B* that resulted in completely disordered motion in agents that do forget (cf. Fig 4a). As one moves to higher densities, the tendency towards long-term collective alignment becomes even more pronounced. Parameters: number of agents *N* = 10, world size *W* = 80, sensory range *r* = 5 (number of bins *B* = 8).

Finally, recall that the learning process of our agents includes a ‘forgetting’ mechanism, which allows them to cope with changing or non-deterministic environments. Fig 7 shows how the results of the learning process are affected if one disables this feature, setting the parameter *γ* = 0. One can see that, in the limit of long learning times, these agents will develop *deterministic* responses to their neighbours’ movement, as the *h*-values associated with rewarded actions grow without bound. As for the resulting collective motion, high decisiveness—as discussed in the previous section—implies long-term alignment even in the limit of low swarm densities. While this might be the optimal solution for a robot navigating a simple artificial environment, it is not what one typically finds in real animals. The reason is that their complex environments generally contain additional features that make deterministic responses inappropriate, for instance adaptable predators, which are best thwarted with unpredictable escape strategies [27]. The ‘forgetting’ parameter in our agents’ learning rules therefore offers not only the possibility of adapting to changing environments [15], but also a compact way of including environmental factors that favour non-deterministic responses.

## Discussion

As a first benchmark of the learning-agent-based model, we tested whether it can reproduce the predictions of existing SPP models. Note that one should not expect this model to predict any new phenomena at this level, since the goal was to gain a better understanding of a given collective dynamics by deriving individual-level interaction rules that can account for it. The results detailed above show, firstly, that our agent-based model can match the achievements of existing SPP models: it can account for the density-dependent switching of group alignment in marching locusts and, at a more quantitative level, for the functional form of the drift and diffusion coefficients in the FPE governing global alignment. (The analysis of the predicted collective dynamics in terms of a FPE is particularly useful for a comparison with other models, as seen in [17].) Indeed, one can see that our agent-based model formally reduces to a conventional SPP model if one disables the learning routine, which ultimately amounts to fixing a set of interaction rules between individuals. However, even in this fixed-interaction limit, agent-based modelling encourages one to develop a more detailed account of the individuals that make up a swarm. This offers some advantages over conventional *ad hoc* SPP models, which we discuss in the following.

In conventional individual-based models, one is generally free to assume that individuals interact according to a set of rules that can take an arbitrary mathematical form—possibly even changing sharply depending on some condition—and that depend on any variables that are represented in the model. By contrast, agent-based models are designed to provide a more natural and realistic (albeit still abstract) account of which perceptions individuals respond to and how these are processed to produce a response. For example, the model presented here introduces parameters that measure how each individual is likely to respond to various configurations of movement of their neighbours, rather than simply imposing that the individual’s speed is deterministically set to some weighted local average broadened by an additive noise term. This difference becomes more pronounced in more complex environments. For example, in a two-dimensional model, agents might be in principle able to perceive the (coarse-grained) positions of all neighbours within some considerable range and learn by themselves to extract the relevant information from this input, rather than being pre-programmed to approach the local centre of mass. Note that the interaction rules that are developed in this process can be verified experimentally, by exposing individuals to the percepts that have been identified as relevant and observing how they respond. Alternatively, when monitoring large populations, without precise control over individuals, one can select those sub-ensembles that happen to encounter circumstances of interest and track their responses, for example with the techniques of [28].

Moreover, our approach favours models whose parameters correspond more closely to independent variables in the real system. This has the convenient consequence of ensuring that theoretical predictions have straightforward operational meaning. However, more importantly, it reduces the risk of predicting spurious effects, which can arise, for example, due to treating certain variables as fundamental when they do, in fact, depend non-trivially on others. A similar type of problem arises if one makes the opposite mistake, of allowing changes in a variable that should, realistically, be fixed: this may lead to overfitting. Such issues are less likely to occur if the parameters of the model are chosen to correspond closely to real independent variables.

To illustrate this point, we begin with an example of the first problem, of treating a parameter as fundamental when it is, in fact, a function of other variables. Consider the two models whose predictions are compared in Fig 3: on the one hand, the model used by Dyson *et al*. [19] reduces the swarm to two populations (moving clockwise and counter-clockwise, respectively) and takes as fundamental parameters the transition rates between these populations. This approach ignores the fact that transition rates are only a coarse-grained description of the effects of distinct individuals receiving various stimuli and responding to them according to their intrinsic dispositions. Consequently, the model cannot predict whether or how the transition rates should change with the number of individuals, *N*, which is normally the independent parameter in the pertinent experiments. By contrast, the basic parameters in our agent-based model describe independently how many individuals there are and how each of them responds to what they perceive, and consequently the model naturally includes a description of how collective effects depend on the population size. In the case of locust marching, in particular, this more careful theoretical treatment should allow us to resolve any disagreements that there may be between experimental observations and existing theoretical models regarding the *N*-dependence of the FPE coefficient functions.

The second problem, overfitting, can be illustrated by the same example: when fitting the transition-rate model to experimental data, the values of the transition rates are found to vary considerably between datasets (see Table 1 in [19]), in a way that is not accounted for by the model. This suggests that the responses of individual locusts would have to vary as a function of density. However, empirical studies show that (once the initial process of behavioural gregarisation is complete) the locusts’ individual-level interactions and activity remain consistent across “a wide range of densities” [21]. For comparison, we implemented this scenario in our agent-based model, fixing the decisiveness *d* (that is, the individual-level interactions) while varying the population size *N* (which, given a world of fixed size, implies varying density). The results in Fig 2 show that our model can reproduce the different regimes of collective motion by varying only the density, in accordance with the above-mentioned empirical findings.

Furthermore, while it is possible to account for the different regimes of collective alignment by varying the density alone, our model also offers the possibility of exploring how the collective dynamics are affected as the underlying individual responses change—not only by manually changing the relevant parameters, which is possible in general individual-based models, but also as part of the adaptive (learning) process. This possibility is particularly intriguing in the context of the transition from the solitarious to the gregarious phase in locusts, which is known to play a central role in the formation of swarms. It would be very interesting to test an agent-based model of locusts in different behavioural phases against experimental observations (which could be obtained, for instance, by exploiting the fact that behavioural changes take some time (on the order of hours [29]) to come into full effect when locusts are crowded).

On the topic of experimental implementations, we note that a move towards more detailed models of the behaviour of individual locusts is particularly timely in light of the advances in data acquisition and analysis that have been made in the last decade. In particular, the methods for automated tracking and analysis of the movement of individual locusts introduced in [21, 28] provide a natural experimental tie-in for agent-based models, since they provide high-volume data on how individuals respond in various scenarios.

A second major advantage of agent-based models is the possibility of agents learning how to respond to their neighbours’ activity based on feedback from the environment, rather than being programmed with a set of interaction rules *ad hoc*. (A similar ansatz, though aimed directly at determining a set of interaction rules, was explored by Guttal *et al*.: they assume an escape-and-pursuit-type interaction [9] and succeed in obtaining the weights of the two interaction terms using genetic algorithms, in a way that models evolutionary adaptation [30].) The idea of using learning agents to determine the rules governing a complex system allows for fundamental improvements in modelling, since it generally reduces the extent of required assumptions, which in turn reduces the risk of undue implicit restrictions or overfitting. The example of marching locusts illustrates how learning-agent-based models require far fewer assumptions than common alternatives: for example, a typical SPP model assumes that individuals adjust their orientation to some linear combination of their neighbours’ mean orientation and possibly an external field—a nontrivial choice of functions, with several parameters that must be specified, such as weights and cutoff radii. By contrast, our agent-based model assumes only that agents receive feedback about the success of their responses in various situations, with no built-in assumptions about the possible ‘correct’ behaviours. One may argue that part of the burden of assumptions is shifted to the specification of the learning problem instead, for instance the specification of the reward function or the parametrisation of the space of available percepts. However, it seems much more enlightening to make assumptions about *why* and *based on what input* individuals respond as they do—even if those assumptions end up proven wrong, in case the agents fail to obtain their rewards or end up exhibiting different dynamics than expected—than to simply postulate that they respond in a certain way.

The results presented in the section ‘Learning individual-level behaviour’ show that agents that are rewarded for aligning themselves with their neighbours learn to respond to their neighbours’ movements in a way that reproduces density-dependent alignment at the swarm level. In a similar way, agents that are presented with a richer perceptual space can autonomously learn which percepts are relevant to them. A simple example of this selection can be seen in Fig 6, which shows that agents come to respond more strongly to percepts that are more relevant to their overall success. Such an investigation, of the kinds of information that must be shared between individuals in order to account for various collective phenomena, is likely to produce concrete predictions that can be tested against feasible experiments, allowing us to further our understanding of the mechanisms that lead to the formation of swarms.

Looking back at the results of this first foray into modelling collective behaviour with agents, one may ask more generally: in which contexts is it helpful—or in fact necessary—to treat the individual constituents of the swarm as agents, in the sense of individuals possessing distinct, changeable behavioural tendencies? The answer is twofold.

For one, it is certainly more appropriate to model individuals as agents when they are in fact too complex to be reduced to a crowd of identical, unchangeable automata. This is most likely the case for humans, or any sufficiently complex animal, which makes agent-based modelling a natural tool for certain parts of behavioural biology or, for example, econophysics. In fact, having provided a rigorous definition of what constitutes an agent, we can make a clear distinction between generic individual-based models and agent-based models in the strict sense. Building on this, one can now *assess* the complexity of swarm behaviour based on whether it can be accounted for by conventional SPP models or whether this requires the new, more general type of model that treats the underlying individuals as full-fledged agents. Naturally, an exploration of the kinds of collective phenomena that can only be understood in terms of agent-based models is beyond the scope of this initial proposal, but they do present an intriguing topic for further research.

In the case of locusts, in particular, one may debate whether it is plausible that individual animals actually learn the behaviours that ultimately give rise to a cohesive swarm. Regarding the learning capabilities of locusts in general, a series of studies have shown that they can change their responses—specifically to olfactory stimuli—by associative learning [31–34]. While these scenarios would make an interesting application of learning-agent-based models in their own right, they provide no evidence as to whether locusts learn interactions with conspecifics in a similar manner. Let us focus, then, specifically on the transition from solitarious to gregarious behaviour: while the early empirical account of Ellis [35] refers to this as a ‘learning’ process, more recent work [36] singles out simple habituation as a key element of behavioural gregarisation. This observation (while relevant to understanding the mechanisms that support phase change) does not address how the behavioural responses that are *modulated* by habituation [37], such as attraction to or avoidance of conspecifics, are acquired in the first place. In summary: while locusts are capable of associative learning, there is no strong evidence to suggest that the interaction rules that lead to swarming are, in fact, learned by individual locusts.

There is, however, a second context in which agent-based modelling is relevant: generally, when studying a species that is well-adapted for survival in a (relatively) simple, predictable environment, it may be reasonable to approximate individuals as automata that are endowed with a fixed set of behavioural rules at birth. However, if one considers instead entire species and the much larger time-scales of evolutionary adaptation, then behavioural tendencies once again become changeable. In the simulations reported here, the PS agents do not represent individual locusts, with limited life-times—instead, they can be taken as avatars carrying the behavioural programmes of entire populations of locusts, which change in response to the environment. This task, of modelling evolutionary adaptation, is a second context in which standard SPP models, with fixed behavioural rules, are inadequate, and agent-based models must be applied.

Since the possibility of learning behaviours is a central feature of agent-based models, the reward scheme which drives this learning process plays an important role. While it may seem more involved, at first sight, to specify what locusts are rewarded for instead of describing directly how they behave, we argue that our reinforcement-based ansatz offers greater insights, since it includes the reasons *why* individuals behave as they do. Moreover, specifying only which achievements are ultimately rewarded ensures that (given sufficiently large sample sizes) the learning agents will discover *all* behaviours that lead to this outcome. This will allow researchers to consider various different possible individual-level behaviours that can account for particular collective phenomena, some of which might have been overlooked in an *ad hoc* approach. For example, even the simple learning task of locust alignment that we consider here already illustrates how different sets of response probabilities—one postulated, the other learned—can lead to practically indistinguishable collective dynamics.

In the present work, one may note that the learning task faced by the agents is rather basic: given a percept that encodes the net movement in the vicinity, an agent is given the choice of either turning around or not and is subsequently rewarded if it successfully aligned itself with its neighbours. Ideally, one should specify a more realistic condition for being rewarded, such as first collecting a large group and subsequently traversing long distances under adverse conditions, in order to test whether cohesion and density-dependent alignment emerge spontaneously from these requirements. While such a problem would make for a more demanding learning task, due to the necessarily larger percept space and highly delayed rewards, this is a natural direction for the next steps in our research. The simpler task considered here is intended only to illustrate that learning agents can improve our understanding of the origins of collective motion *in principle*. In light of the results discussed above, we now look forward to combining our ansatz with more realistic survival tasks, based on insights from biology and ecology, in order to explore the phenomenon of collective motion in more complex settings.

## Supporting information

S1 Appendix From learning agents to a Fokker-Planck equation. Derivation of the collective dynamics of learning agents in terms of a FPE.
(PDF)

S1 Fig Stochastic transition functions *P*(*z*′|*z*) describing the evolution of (the probability distribution over) the global alignment parameter, for the agents with fixed interaction rules described in Eq (3) in the main text. Parameters: *N* = 100, (a) *d* = 1, *B* = 1000, (b) *d* = 1, *B* = 100, (c) *d* = 1, *B* = 10, (d) *d* = 10, *B* = 1000, (e) *d* = 10, *B* = 100, (f) *d* = 10, *B* = 10.
(EPS)
