# Supplementary material for: Modelling collective motion based on the principle of agency: General framework and the case of marching locusts
Source: PLoS One. 2019 Feb 20;14(2):e0212044. doi: 10.1371/journal.pone.0212044 (PMC6382133; doi:10.1371/journal.pone.0212044)
Supplement: S1 Appendix — Derivation of the collective dynamics of learning agents in terms of a FPE. (PDF) [file pone.0212044.s001.pdf]

Modelling collective motion based on the principle of agency: general framework and the case of marching locusts

Katja Ried[1*], Thomas Müller[2], Hans J. Briegel[1,2]

1 Institut für Theoretische Physik, Universität Innsbruck, Technikerstraße 21a, 6020 Innsbruck, Austria
2 Department of Philosophy, University of Konstanz, 78457 Konstanz, Germany

* Katja.Ried@uibk.ac.at

Appendix: From learning agents to a Fokker-Planck equation

This appendix begins with the agent-based model presented in the main paper and derives a continuous-variable description of the group dynamics, in the form of a Fokker-Planck equation for the probability density $P(z,t)$. The derivation proceeds as follows: We begin by focusing on a single (generic) individual and computing the probabilities that this individual receives different percepts, given the current value of the global alignment parameter. Combining this information with the conditional probabilities for turning or continuing in the same direction, which are derived from the h-matrix of the PS model, one can then compute the probability that a given individual will turn around in the current time-step. From this, in turn, one can in general compute how many individuals convert from going in one direction to the opposite, and consequently the global alignment of the population in the next time-step. Since the entire process is stochastic, this yields a probability distribution over z at the next time-step. Taking the limit in which the time t and the alignment parameter z become continuous, the dynamics can be cast as a Fokker-Planck equation, whose drift and diffusion coefficients we derive as a function of – ultimately – the h-matrix, which describes the memory of the individual locust. This form will allow us to compare the predictions of our model to the results of other works.

Derivation of group-level transition probabilities

We begin by establishing the relevant variables. In addition to the total number of agents N, we will use the combination $B = W/2r$, which specifies how many neighbourhoods (or 'bins', defined as regions that an individual can see) the world is divided into. (Recall that r denotes the sensory range, i.e., the distance up to which a given agent can perceive others, while W is the size W of the world.) For the first part of the derivation, which is cast explicitly in terms of discrete individuals, it is convenient to use the variable X_+ denoting the number of individuals moving in the clockwise direction (which we arbitrarily label as positive) at the beginning of the current time-step, while $X_- = N - X_+$ individuals are moving anticlockwise. This information can equivalently be expressed by the alignment parameter $z = \frac{1}{N}(X_+ - X_-)$.

Focusing on a single, generic agent, the *focal agent*, let $\tilde{X}_\pm$ denote the numbers of individuals moving in each direction within this agent's sensory range, while $\epsilon = \pm 1$ indicates in which direction the agent itself is moving. Thus, $\epsilon\left(\tilde{X}_+ - \tilde{X}_-\right)$ is the net

flow of neighbours relative to the focal agent. Since the agent only distinguishes absolute values up to two, the percept s can be written as

$$s = \mathrm{trunc}\left[\epsilon\left(\tilde{X}_+ - \tilde{X}_-\right)\right] \in \{-2, -1, 0, +1, +2\}\,. \tag{1}$$

Based on the probabilities of a given individual turning around, we will compute the number of individuals turning from positive to negative (clockwise to anticlockwise), denoted D_-, and the number turning the opposite way, denoted D_+. The variable of interest in this calculation is the number of individuals moving clockwise in the subsequent time-step, $X'_+ = X_+ + (D_+ - D_-)$, or equivalently the change $\Delta X_+ = D_+ - D_-$.

Probability distribution over percepts. We begin by determining the probability that the focal agent receives a certain percept, which is essentially the difference between the numbers of individuals moving each way within its sensory range, $\tilde{X}_+ - \tilde{X}_-$. In order to derive a tractable expression for this quantity, we make three assumptions:

- In order to derive transition probabilities that depend only on the fraction of individuals going each way but not on their individual positions, we will assume that the individuals are approximately homogeneously distributed in space.
- Moreover, we assume that individuals are *independently* distributed in space. (This assumption is inaccurate in the limit of low densities, when, according to our simulations, individuals tend to congregate in groups with density $\approx 1/r$. However, as soon as the number of agents relative to the size of the world is high enough, $N/W > 1/r$, it becomes reasonable to assume a homogeneous, independent distribution.)
- Finally, we will neglect the fact that the overall number of individuals going in each way, $X_\pm$, is in fact finite. This is justified by different considerations, depending on the regime: If $B \gg 1$, then only a small fraction of the total $X_\pm$ is located within a given bin. Therefore, for the purpose of determining how many individuals are in this bin, the approximation that one is drawing from an infinite pool is reasonable. If, however, $B \to 1$, then the number $\tilde{X}_\pm$ of individuals moving in a given direction within the focal agent's sensory range is most likely large anyway (of order N), and since percepts only distinguish $\tilde{X}_+ - \tilde{X}_-$ up to absolute values of 2, overestimating these numbers is unlikely to cause deviations.

Under these assumptions, the numbers $\tilde{X}_\pm$ follow a Poisson distribution with mean $X_\pm/B$. (One may note that the means of the distributions $P\left(\tilde{X}_\pm\right)$ are related by the constraint $X_+ + X_- = N$. However, the particular values for $\tilde{X}_\pm$ that we draw from these distributions – that is, how many of the $X_\pm$ individuals going each way are within the focal agent's sensory range – are statistically independent. (In fact, this statistical independence also holds for finite N, in which case $\tilde{X}_\pm$ follow binomial distributions.) The difference between two Poisson-distributed variables with means $\mu_{1,2}$ follows a Skellam distribution,

$$\mathrm{Sk}\left(s'; \mu_1, \mu_2\right) \equiv \mathrm{e}^{-(\mu_1+\mu_2)} \left(\frac{\mu_1}{\mu_2}\right)^{\frac{s'}{2}} I_{s'}\left(2\sqrt{\mu_1\mu_2}\right), \tag{2}$$

where $I_{s'}(z)$ denotes the modified Bessel function of the first kind. In our case, this gives the probability distribution over s' conditioned on (i.e., if one knows the value of)

the total number of individuals moving in the positive direction, X_+:

$$P\left(\tilde{X}_+ - \tilde{X}_- = s'|X_+\right) = \mathrm{Sk}\left(s'; \frac{X_+}{B}, \frac{X_-}{B}\right)$$
$$= \mathrm{e}^{-N/B}\left(\frac{X_+}{X_-}\right)^{\frac{s'}{2}} I_{s'}\left(\frac{2}{B}\sqrt{X_+X_-}\right), \tag{3}$$

where one can replace $X_- = N - X_+$. (Note that we condition only on X_+, since including X_- in the known information would be redundant.)

Notice that the difference $\tilde{X}_+ - \tilde{X}_- = s'$ can in principle run over all integers; only in the agent's perception are values $s' \geq 2$ resp. $s' \leq -2$ combined into a single percept each. The sum

$$P\left(\tilde{X}_+ - \tilde{X}_- \geq 2|X_+\right) = \mathrm{e}^{-N/B}\sum_{s'=2}^{+\infty}\left(\frac{X_+}{N-X_+}\right)^{\frac{s'}{2}}$$
$$\cdot I_{s'}\left(2\sqrt{X_+\left(N-X_+\right)}\right), \tag{4}$$

is simply an element of the cumulative density function associated with the Skellam distribution, and analogously $P\left(\tilde{X}_+ - \tilde{X}_- \leq -2|X_+\right)$. If the focal agent is currently moving in the positive (clockwise) direction, then this probability distribution is precisely the probability distribution over percepts, $s = s'$ (with the cutoff $|s| \leq 2$), whereas for agents moving in the negative direction, the percept is $s = \tilde{X}_- - \tilde{X}_+ = -s'$ (again enforcing $|s| \leq 2$).

Probability of turning. In the PS model, the h-matrix determines the probabilities of turning around given a percept, $P\left(turn|s\right)$. Together with the probabilities computed in the previous section, $P\left(s|X_+, \epsilon\right)$, this allows us to obtain

$$P\left(turn|X_+, \epsilon = \pm\right) = \sum_s P\left(turn|s\right) P\left(s|X_+, \epsilon\right), \tag{5}$$

which indicate how likely it is that a particular individual from either sub-population (clockwise or anticlockwise) turn around in a given time-step. Notice that, under the assumptions detailed above, this probability depends only on the global variable X_+ and the individual's orientation ϵ.

Probability distribution over populations $X'_\pm$. In order to obtain the populations going in each direction at the next time-step, $X'_\pm$, we will now compute the numbers of individuals turning from the positive to the negative direction (clockwise to anticlockwise), denoted D_-, and the number turning from negative to positive, denoted D_+. Notice that only the difference $D_+ - D_-$ manifests as an effective increase of the population X_+, but for the purpose of computing transition probabilities, one must distinguish how many individuals turned in each direction, even if their numbers partially cancel afterwards.

Since the individuals that turn, $D_\pm$, are necessarily a subset of those currently moving in the direction in question, $X_\mp$, we model $D_\pm$ as following a binomial distribution:

$$P\left(D_\pm|X_+\right) = \mathrm{Binom}\left(D_\pm; X_\mp, p = Prob(turn|\mp)\right)$$
$$= \begin{pmatrix} X_\mp \\ D_\pm \end{pmatrix} p^{D_\pm}\left(1-p\right)^{\left(X_\mp - D_\pm\right)}. \tag{6}$$

The probability distribution over $\Delta X_+ = D_+ - D_-$ resulting from this model does not have a closed form, requiring instead a sum over the various combinations of $D_\pm$ that lead to each ΔX_+. Specifically, letting $\mathcal{D}$ denote the set of ordered pairs (D_+, D_-) such that $D_+, D_- \geq 0$ and $\Delta X_+ = D_+ - D_-$, we can write

$$P\left(\Delta X_+ | X_+\right) = \sum_{(D_+, D_-) \in \mathcal{D}} P\left(D_+ | X_+\right) P\left(D_- | X_+\right). \tag{7}$$

Result: discrete-time transition matrices. The above method allows one to derive a stochastic rule that describes the changes to the probability distribution over the alignment parameter. In the discrete case, $P\left(X_+\right)$ can be represented as a vector, and the transition probabilities $P\left(X'_+ | X_+\right)$ take the form of a matrix. In the limit of large population size N, it is convenient to replace the argument X_+ by $z = \frac{1}{N}\left(X_+ - X_-\right)$, which becomes continuous as $N \to \infty$, making the distribution $P\left(z\right)$ and the conditional $P\left(z'|z\right)$ functions of one resp. two continuous variables, with range $[-1, 1]$. The conditional probability $P\left(z'|z\right)$ specifies how the probability distribution $P\left(z\right)$ changes in a time-step Δt (that is, during the time it takes for an agent to deliberate and choose its next action), and it allows one to read off key features of the collective dynamics, such as whether there are metastable states, how strongly aligned the group is in these states, and how quickly the system transitions between them.

The transition functions generated by our model, with the fixed h-matrix given by Eq. (3) in the main text, are shown in Fig. A1. All instances exhibit a narrow band of non-negligible probabilities, which implies that the mapping from z at time t to z' at time $t + \Delta t$ is approximately deterministic. If this band lies in the diagonal $z' = z$, then the alignment parameter tends to remain unchanged at any value. Fig. A1 shows how the dynamics deviates from this default in response to two parameters: Firstly, as the effective density N/B increases, the peak of $P\left(z'|z\right)$ remains at a fixed, large $|z'|$ for a wider range of z. That is, the dynamics maps a wider range of intermediate states z to a particular pair of strongly aligned states. This can be understood as a consequence of high densities quickly suppressing non-aligned states. Secondly, as decisiveness d increases, the value of $|z'|$ to which the system tends increases; that is, the two metastable states between which the system alternates become more strongly aligned. This can be attributed to the high decisiveness making individuals less likely to turn against the majority. (One can see this effect clearly in the following example: if the group was initially perfectly aligned, $z = 1$, then the expected alignment at the next time-step is $z' = 1 - \frac{1}{1+d/2}$.)

Making the alignment parameter z and the time t continuous

The limit of continuous time and transition rates. The model derived above gives the probabilities of finite changes in the populations $X_\pm$ over discrete and finite time-steps Δt, which is natural in the context of reinforcement learning. However, in order to relate our work to other models that may not necessarily assume discrete time, we will now modify the above treatment to recover continuous time. To this end, we will introduce an infinitesimally small interval δt and determine the transition probabilities $P_{\delta t}\left(X'_+ | X_+\right)$ for this time-step.

In order to derive group-level transition probabilities $P_{\delta t}\left(X'_+ | X_+\right)$ for an infinitesimally small δt, we begin with the following consideration: if a single individual has a probability $P_{\Delta t}\left(turn|s\right)$ of turning around in a finite time interval of default duration Δt, then the probability of turning in a smaller time interval δt is proportionately smaller, $\frac{\delta t}{\Delta t} P_{\Delta t}\left(turn|s\right)$. Formally, this assertion can be derived from the assumption that 'turning' is an instantaneous event that could happen with uniform probability at any time. The probability of not turning, which is the absence of such an

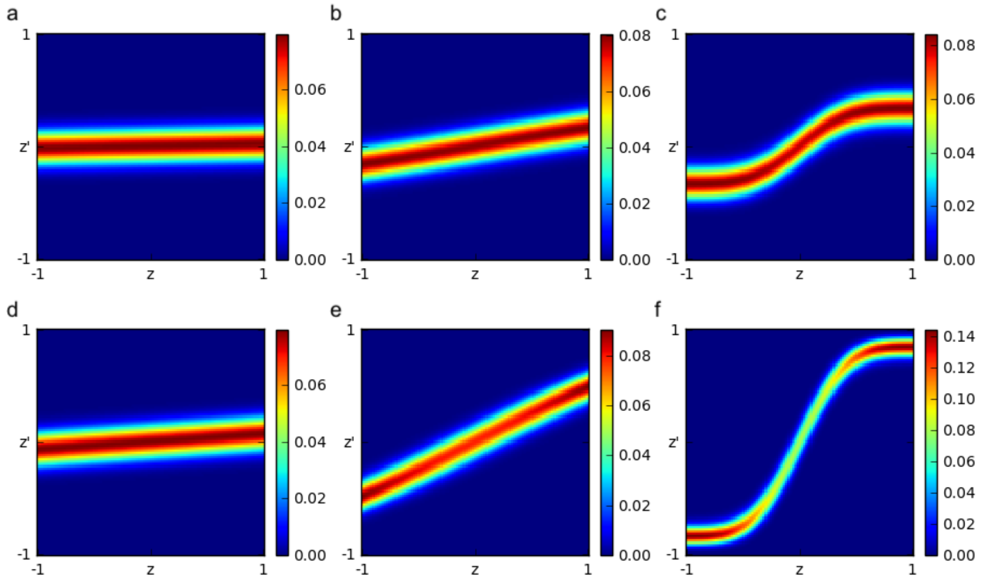

Fig A1. Stochastic transition functions $P\left(z'|z\right)$ describing the evolution of (the probability distribution over) the global alignment parameter, for the agents with fixed interaction rules described in Eq. (3) in the main text. Parameters: $N = 100$, (a) $d = 1$, $B = 1000$, (b) $d = 1$, $B = 100$, (c) $d = 1$, $B = 10$, (d) $d = 10$, $B = 1000$, (e) $d = 10$, $B = 100$, (f) $d = 10$, $B = 10$. (Note that the independent parameters W and r do not appear in the calculations leading to these plots; only the ratio $B = W/2r$ matters.)

event, is consequently $1 - P_{\Delta t}\left(turn|s\right)$ and $1 - \frac{\delta t}{\Delta t} P_{\Delta t}\left(turn|s\right)$, respectively. This prescription changes the probabilities (5) of turning and staying that we derive from the h-matrix (which is based on the finite Δt), giving

$$\begin{aligned} &P_{\delta t}\left(turn|X_+, \epsilon = \pm\right) \\ &\quad= \sum_s \frac{\delta t}{\Delta t} P_{\Delta t}\left(turn|s\right) P\left(s|X_+, \epsilon\right). \end{aligned} \tag{8}$$

It will be convenient to define transition *rates* (probability per time) for a given individual in a particular sub-population turning around, given the current value of X_+:

$$\begin{aligned} \tau_{\pm}\left(X_+\right) &\equiv \lim_{\delta t \to 0} \frac{P_{\delta t}\left(turn|X_+, \epsilon = \mp\right)}{\delta t} \\ &= \sum_s \frac{P_{\Delta t}\left(turn|s\right)}{\Delta t} P\left(s|X_+, \epsilon = \mp\right). \end{aligned} \tag{9}$$

This allows us to write the probabilities as

$$P_{\delta t}\left(turn|X_+, \epsilon = \pm\right) = \delta t \cdot \tau_{\mp}\left(X_+\right), \tag{10}$$

thereby making the dependence on the time interval explicit.

The remainder of the derivation of $P\left(\Delta X_+|X_+\right)$ proceeds as before, resulting in a combination of two binomial distributions. For notational simplicity, let us restrict ourselves to the case $\Delta X_+ \geq 0$. (The alternative case follows by exchanging $+$ and $-$.) Recalling that $\mathcal{D}$ is the set of (D_+, D_-) such that $D_+, D_- \geq 0$ and $\Delta X_+ = D_+ - D_-$, this implies that we must sum over $D_+ \geq \Delta X_+$. On the other hand, since the

individuals turning into the positive direction are drawn from X_-, it holds that $D_+ \leq X_-$. This gives

$$\begin{aligned} &P_{\delta t}\left(\Delta X_+ | X_+\right) \\ &\quad = \sum_{D_+ = \Delta X_+}^{X_-} \begin{pmatrix} X_- \\ D_+ \end{pmatrix} \begin{pmatrix} X_+ \\ D_+ - \Delta X_+ \end{pmatrix} \\ &\quad \cdot \left[\delta t \tau_+\right]^{D_+} \left[1 - \delta t \tau_+\right]^{(X_- - D_+)} \\ &\quad \cdot \left[\delta t \tau_-\right]^{(D_+ - \Delta X_+)} \left[1 - \delta t \tau_-\right]^{(X_+ - D_+ + \Delta X_+)} . \end{aligned} \tag{11}$$

As soon as δt becomes small enough that $\tau_\pm \delta t \ll 1$ – in other words, that the turning probabilities for a single individual become small –, one can neglect the terms with large numbers D_+ and $D_- = D_+ - \Delta X_+$ of individuals turning in both directions. Assuming that this makes $D_\pm \ll X_\mp$, one can then approximate the binomial coefficients as $X_\pm^{D_\mp} / \left(D_\mp !\right)$, yielding

$$\begin{aligned} P_{\delta t}\left(\Delta X_+ | X_+\right) \approx & \sum_{D_+ \geq \Delta X_+} \frac{1}{D_+! \left(D_+ - \Delta X_+\right)!} \\ & \cdot \left[X_- \delta t \tau_+\right]^{D_+} \left[X_+ \delta t \tau_-\right]^{(D_+ - \Delta X_+)} . \end{aligned} \tag{12}$$

If one suppresses all but the lowest order in $X_\mp \delta t \tau_\pm$, which means neglecting all terms except $D_+ = \Delta X_+$, one obtains

$$P_{\delta t}\left(\Delta X_+ | X_+\right) \approx \begin{cases} \frac{1}{\Delta X_+!} \left[X_- \delta t \tau_+\right]^{\Delta X_+} & \Delta X_+ \geq 0 \\ \frac{1}{\Delta X_+!} \left[X_+ \delta t \tau_-\right]^{\Delta X_+} & \Delta X_+ \leq 0 \end{cases} . \tag{13}$$

One can see that the conditional probability $P_{\delta t}\left(X_+' | X_+\right)$ becomes sharply peaked around $X_+' = X_+$, with small values for $X_+' = X_+ \pm 1$ and negligible values outside that region. That is, the most relevant quantities are

$$P_{\delta t}\left(X_+' = X_+ \pm 1 | X_+\right) \approx \delta t \cdot X_\mp \tau_\pm \left(X_+\right) . \tag{14}$$

Again, for notational simplicity, we introduce the transition rates

$$T_\pm \left(X_+\right) \equiv \lim_{\delta t \to 0} \frac{P_{\delta t}\left(X_+' = X_+ \pm 1 | X_+\right)}{\delta t} . \tag{15}$$

Substituting the approximate expression for $P_{\delta t}\left(X_+' = X_+ \pm 1 | X_+\right)$,

$$T_\pm \left(X_+\right) \approx X_\mp \tau_\pm \left(X_+\right) . \tag{16}$$

As one should expect, the probability of any one out of $X_\mp$ individuals turning around grows linearly as one increases the number of individuals one is sampling from. This observation will become relevant in the next section.

The limit of continuous alignment parameter z. The final step is to make the alignment parameter $z \equiv \frac{2X_+}{N} - 1$ continuous, by letting $N \to \infty$. To this end, notice that one can rewrite the transition rates obtained in the previous subsection as

$$T_\pm (z) \equiv \lim_{\delta t \to 0} \frac{P_{\delta t}\left(z' = z \pm \frac{2}{N} | z\right)}{\delta t} . \tag{17}$$

In the limit of continuous time, as simultaneous transitions of more than one individual become negligibly unlikely, the balance of probabilities of (a) leaving the state with a particular z and (b) reaching that state starting from nearby states with $z' = z \pm \frac{2}{N}$ is

$$\begin{aligned}\frac{\partial}{\partial t} P\left(z,t\right) = &-\left[T_{+}\left(z\right) + T_{-}\left(z\right)\right] P\left(z,t\right) \\ &+T_{+}\left(z-\frac{2}{N}\right) P\left(z-\frac{2}{N},t\right) \\ &+T_{-}\left(z+\frac{2}{N}\right) P\left(z+\frac{2}{N},t\right). \end{aligned} \tag{18}$$

One can now use a Taylor expansion around z to rewrite this as

$$\begin{aligned}\frac{\partial}{\partial t} P\left(z,t\right) = &-\frac{2}{N}\frac{\partial}{\partial z}\left[\left\{T_{+}\left(z\right) - T_{-}\left(z\right)\right\} P\left(z,t\right)\right] \\ &+\frac{4}{2N^2}\frac{\partial^2}{\partial z^2}\left[\left\{T_{+}\left(z\right) + T_{-}\left(z\right)\right\} P\left(z,t\right)\right] + \mathcal{O}\left(\frac{1}{N^3}\right). \end{aligned} \tag{19}$$

This has the form of a Fokker-Planck equation,

$$\frac{\partial}{\partial t} P\left(z,t\right) = -\frac{\partial}{\partial z}\left[F\left(z\right) P\left(z,t\right)\right] + \frac{\partial^2}{\partial z^2}\left[D'\left(z\right) P\left(z,t\right)\right], \tag{20}$$

with coefficients

$$\begin{cases} F\left(z\right) = \frac{2}{N}\left[T_{+}\left(z\right) - T_{-}\left(z\right)\right] \\ D'\left(z\right) = \frac{4}{2N^2}\left[T_{+}\left(z\right) + T_{-}\left(z\right)\right]. \end{cases} \tag{21}$$

One can verify that, as one takes the continuous limit, $F(z)$ is simply the drift coefficient, while $D'(z)$ is closely related to the diffusion coefficient $D(z)$ introduced in the main article.

It is instructive to rewrite these expressions in terms of more fundamental quantities, in particular the transition rates $\tau_{\pm}$ for single individuals, defined in Eq. (9). Using the simplified expression for $T_{\pm}$ from Eq. (16) and recalling that $X_{\pm} = \frac{N}{2}\left(1 \pm z\right)$, one can write

$$\begin{cases} F\left(z\right) \approx \left(1-z\right)\tau_{+}\left(z\right) - \left(1+z\right)\tau_{-}\left(z\right) \\ D\left(z\right) \approx \frac{1}{N}\left[\left(1-z\right)\tau_{+}\left(z\right) + \left(1+z\right)\tau_{-}\left(z\right)\right]. \end{cases} \tag{22}$$

One can see that the pre-factor $\frac{2}{N}$ in the expression for $F\left(z\right)$, which appeared as a by-product of the first derivative ∂_z, is cancelled by the fact that the group-level transition rates $T_{\pm}$ increase linearly with N. Consequently, the function $F\left(z\right)$ – when written in terms of the individual transition probabilities $\tau_{\pm}$ – remains finite in the limit of infinitely many individuals, and gives the drift coefficient.

The coefficient $D'(z)$, on the other hand, acquires a pre-factor $\frac{4}{N^2}$ due to the second derivative ∂_z^2, which is only partially cancelled by the N-scaling of the transition rates $T_{\pm}$. Consequently, when written in terms of the individual transition rates $\tau_{\pm}$, it vanishes as $1/N$ in the limit of infinitely many individuals. This is, in fact, not surprising. To see this, consider the example of N completely non-interacting individuals, each of which independently changes direction with some fixed transition rate τ: as a function of N, the number of individuals that change direction per time interval grows as N, but the net change in the number of aligned individuals – since most of the changes cancel out – scales only as $\sqrt{N}$. (It is equivalent to the expected traversed distance in an N-step random walk.) Moreover, changing the number of aligned individuals by 1 only changes the normalised parameter z by $2/N$. Thus, the overall change to z over a fixed time scales as $1/\sqrt{N}$, and the diffusion coefficient, which

is related to the square of this parameter, shrinks as $1/N$ with the size of the population. The object of interest for the present work, however, is how the diffusion varies *in addition* to this scaling, and in order to isolate this effect, it is preferable to consider

$$D(z) \equiv N D'(z)\,. \tag{23}$$

One can verify that, if one keeps the decisiveness d and the density N/B constant while varying the number of individuals, then this $D(z)$ remains unchanged.

Finally, we note that, in the extrema $z = \pm 1$, the expressions for the drift and the diffusion coefficients reduce to

$$\begin{cases} F(z=-1) = \tau_+(z=-1) = D(z=-1) \\ -F(z=+1) = \tau_-(z=+1) = D(z=+1)\,. \end{cases}$$

One can see this relation between drift and diffusion in the predictions of our model, shown in Fig. 3a in the main text. More importantly, the same can be seen in Fig. 3c, which depicts the predictions of a model that Dyson *et al.* fitted to experimental data.
